# Adenosine triphosphate induces amorphous aggregation of amyloid β by increasing Aβ dynamics

**DOI:** 10.1038/s41598-024-58773-6

**Published:** 2024-04-07

**Authors:** Masahiro Kuramochi, Momoka Nakamura, Hiroto Takahashi, Tomoe Komoriya, Teisuke Takita, Ngan Thi Kim Pham, Kiyoshi Yasukawa, Kazuaki Yoshimune

**Affiliations:** 1 Graduate School of Science and Engineering, Ibaraki University, Hitachi, 316-8511 Japan; 2 Department of Applied Molecular Chemistry, Graduate School of Industrial Technology, Nihon University, 1-2-1, Izumichou, Narashino, Chiba 275-8575 Japan; 3 Department of Sustainable Engineering, College of Industrial Technology, Nihon University, 1-2-1, Izumichou, Narashino, Chiba 275-8575 Japan; 4 Division of Food Science and Biotechnology, Graduate School of Agriculture, Kyoto University, Sakyo-ku, Kyoto, 606-8502 Japan

**Keywords:** Biological physics, Alzheimer's disease

## Abstract

Amyloid β (Aβ) aggregates into two distinct fibril and amorphous forms in the brains of patients with Alzheimer’s disease. Adenosine triphosphate (ATP) is a biological hydrotrope that causes Aβ to form amorphous aggregates and inhibit fibril formation at physiological concentrations. Based on diffracted X-ray blinking (DXB) analysis, the dynamics of Aβ significantly increased immediately after ATP was added compared to those in the absence and presence of ADP and AMP, and the effect diminished after 30 min as the aggregates formed. In the presence of ATP, the β-sheet content of Aβ gradually increased from the beginning, and in the absence of ATP, the content increased rapidly after 180 min incubation, as revealed by a time-dependent thioflavin T fluorescence assay. Images of an atomic force microscope revealed that ATP induces the formation of amorphous aggregates with an average diameter of less than 100 nm, preventing fibrillar formation during 4 days of incubation at 37 °C. ATP may induce amorphous aggregation by increasing the dynamics of Aβ, and as a result, the other aggregation pathway is omitted. Our results also suggest that DXB analysis is a useful method to evaluate the inhibitory effect of fibrillar formation.

## Introduction

One of the major hallmarks of Alzheimer’s disease (AD) is the formation of senile plaques that primarily contain amyloid β (Aβ) fibrils within the brain. Before the senile plaque formation, diffuse plaques of amorphous Aβ aggregates are found in the brains of individuals with AD^1^. The fibril forms a β-sheet structure, and the amorphous aggregates are formed from the most toxic oligomers by pathways that are on- and off-pathways, respectively. Their toxicity differs in various reports, probably due to the distinct preparation conditions, which determine the aggregation forms^2–4^. Regardless, the on- and off-pathway aggregates show distinct toxicity, and controlling aggregation to decrease toxicity can be an effective therapy for AD. However, evaluating aggregates is challenging, especially at the early stages, due to the instability of the Aβ monomer and the aggregates.

Several Aβ binding molecules have been reported to reduce oligomer toxicity by accelerating the oligomer into the on-pathway and off-pathway aggregates^5–8^. Adenosine triphosphate (ATP) is a hydrotrope that solubilizes hydrophobic molecules at physiological concentrations between 5 and 10 mM^9^. Aβ misfolding is prevented by ATP at lower concentrations, i.e., 0.5 mM^10^. Molecular dynamics simulations suggest that the hydrophobic adenosine of ATP interacts with Aβ and that ATP converts the oligomer into the off-pathway^11^. Furthermore, ATP concentrations decrease in the neocortex of AD patients as AD progresses, suggesting a molecular link between ATP and Aβ^12^. However, the detailed mechanisms by which ATP induces the off-pathway are unclear.

Diffracted X-ray blinking (DXB) monitors the rotational motions of single protein molecules labeled with gold nanocrystals^13^. DXB differentiates the dynamics of Aβ_42_, Aβ_40,_ and Aβ_38_ on a subnanometer scale and reveals that Aβ_42_ possesses lower dynamics among the isoforms, especially after 96 h incubation^14^. Because Aβ_42_ exhibits a higher tendency to aggregate among the isoforms due to the C-terminal hydrophobic amino acid residues, the decrease in dynamics may represent the stacked molecules on the surface of aggregates. Thioflavin T (ThT) selectively interacts with the β-sheet structure in the fibril forms of Aβ and exhibits an increase in fluorescence^15^. Thus, ThT is often used as a fluorescent marker for fibril forms of Aβ. Large amorphous aggregates partially contain β-sheet structures, which are detected by ThT^16^. Off-pathway aggregates with β-sheet structures distinct from the on-pathway fibrils have also been reported^3^. Here, the effect of ATP on the aggregation of Aβ_42_ was evaluated by monitoring the dynamics of Aβ_42_. The increased dynamics of Aβ_42_ may explain the distinct aggregation form induced by ATP.

## Results

### *Dynamic behaviors of Aβ*_*42*_

**Figure 1:**
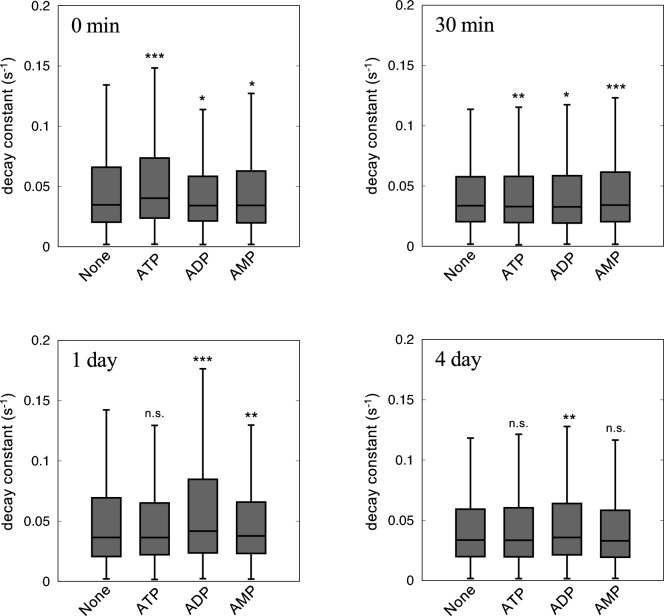
Box plots of the decay constants. Aβ was incubated at 37 °C in the presence or absence of 10 mM ATP, ADP, or AMP and observed by DXB measurement. The distributions of data in the absence were compared with the absence: ***q < 0.001, **q < 0.01, *q < 0.05, n.s. not significant.

To investigate the dynamic behaviors of Aβ_42_ in response to the addition of ATP, we obtained DXB measurements to monitor the subnanometer rotational motions of Aβ_42_. Aβ samples for DXB analysis were prepared based on the method described in a previous study^14^ with some modifications (Fig. S1A). DXB analyses the dynamical fluctuations of each Aβ_42_ molecule by calculating the decay constant of the autocorrelation function from the blinking behavior of the diffraction spots (Figs. S1 and S2). Higher decay constant values indicate higher fluctuation of Aβ molecules (Figs. S2 and S3). The addition of ATP resulted in a particularly significant increase in the 0 min decay constant (Fig. 1A), though less than 3 mM ATP showed no significant effect (Table S1). This result corresponded with the knowledge that ATP functions as a hydrotrope to increase the solubility of hydrophobic Aβ_42_^9^. This effect diminished after 30 min of incubation, suggesting that Aβ_42_ formed metastable aggregates within 30 min. The median value of the decay constants increased after 1 day of incubation and decreased after 4 days of incubation (Table 1 and Fig. S4). The decreased values may indicate that the aggregates are in a metastable state, as described previously^14^. The distribution of decay constants was clustered using a Gaussian mixture model, revealing three main components in the distribution (Fig. S5, Table 1). The 2nd component after 1 day of incubation may represent the instability of Aβ_42_ aggregates, except for that in the presence of ATP.

**Table 1:** Statistical analysis of the decay constant values of Aβ.

Condition		Median	Main comp	2nd comp	3rd comp	q-value*	n**
0 min	None	0.0347	0.0255 (60%)	0.0731 (30%)	0.2379 (10%)	–	9992
	ATP	0.0402	0.0310 (65%)	0.0946 (28%)	0.2792 (7%)	2.2 × 10^−16^	7014
	ADP	0.0342	0.0276 (67%)	0.0735 (24%)	0.2128 (8%)	0.02724	11,570
	AMP	0.0343	0.0259 (63%)	0.0749 (28%)	0.2515 (8%)	0.02744	13,603
30 min	None	0.0326	0.0257 (66%)	0.0719 (27%)	0.2327 (8%)	–	8822
	ATP	0.0317	0.0253 (68%)	0.0731 (24%)	0.2353 (8%)	0.006605	8248
	ADP	0.0338	0.0265 (66%)	0.0741 (26%)	0.2316 (8%)	0.01694	8732
	AMP	0.0353	0.0275 (65%)	0.0779 (27%)	0.2560 (8%)	1.327 × 10^−9^	8078
1 day	None	0.0365	0.0254 (60%)	0.0731 (30%)	0.2379 (10%)	–	10,648
	ATP	0.0364	0.0310 (65%)	0.0946 (28%)	0.2792 (7%)	0.9903	11,854
	ADP	0.0418	0.0276 (68%)	0.0735 (24%)	0.2128 (8%)	2.2 × 10^−16^	4355
	AMP	0.0378	0.0259 (63%)	0.0749 (28%)	0.2515 (8%)	0.003138	16,180
4 day	None	0.0336	0.0262 (67%)	0.0770 (26%)	0.2532 (7%)	–	15,753
	ATP	0.0335	0.0260 (66%)	0.0766 (26%)	0.2439 (8%)	0.7838	15,007
	ADP	0.0345	0.0271 (66%)	0.0815 (26%)	0.2588 (8%)	0.003392	7819
	AMP	0.0336	0.0266 (67%)	0.0775 (26%)	0.2458 (7%)	0.624	11,144

Parentheses represent existence ratios in the conditions.

*The q-values were calculated between the value in the absence (None) and the presence of nucleotides (ATP, ADP, and AMP).

**The number of obtained data.

### Effect of nucleotides on fibril formation

**Figure 2:**
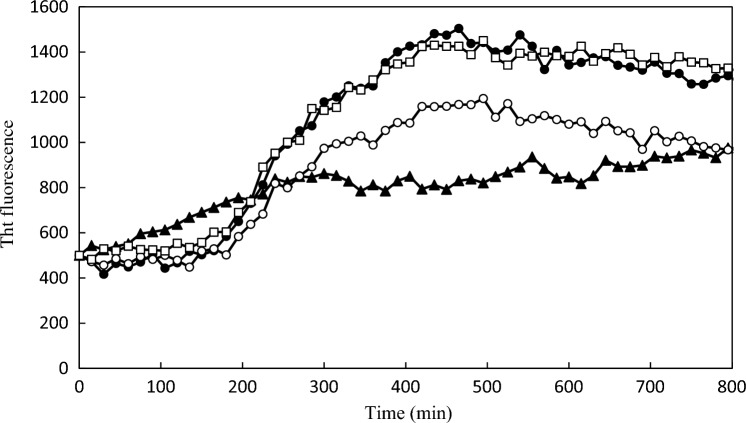
Fibril formation monitored by ThT. Formation of Aβ aggregates in the presence of 10 mM ATP (Filled triangle), ADP (Open circle) or AMP (Open square) and the absence (Filled circle) were monitored by ThT fluorescence.

**Figure 3:**
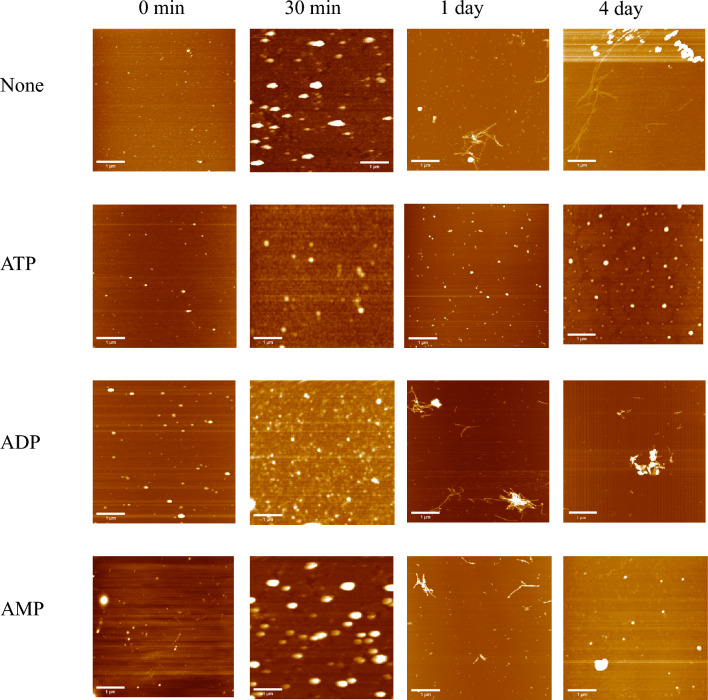
AFM images. Aβ aggregates formed in the presence or absence of 10 mM ATP, ADP, or AMP were observed by AFM.

The Aβ monomer was prepared from 26-*O*-acyliso-Aβ_42_ (iso-Aβ), which starts aggregating immediately after dissolving in an aqueous solution^17^. The β-sheet content of the formed aggregates increased for days based on CD spectra (Fig. S6). The fibril formation of iso-Aβ was monitored using the ThT fluorescence assay. ThT fluorescence rapidly increased after 150 min incubation in the absence of ATP (Fig. 2). This result corresponds with the knowledge that nucleus formation during conversion into the fibril form is a rate-limiting step, and the subsequent elongation of fibrils proceeds faster^18^. The addition of ATP altered the time course of fluorescence and gradually increased the fluorescence. This gentle increase in fluorescence suggests that ATP induces the formation of Aβ_42_ aggregates with β-sheet structures, similar to polyphenolic flavonoids^6^. Atomic force microscopy (AFM) analysis revealed that ATP induced the formation of amorphous aggregates with an average diameter of less than 100 nm (Fig. 3) analyzed using ImageJ^19^. The average diameter of aggregates after 4 days of incubation was smaller in the presence of ATP than in the absence of ATP. Furthermore, ATP seemed to prevent fibril formation since no fibril formation was observed in the tested conditions. The formed amorphous aggregates may be metastable aggregates, including β-sheets, which resulted in the increase in the fluorescence of ThT since amorphous aggregates often partially include β-sheet structures^16^. ATP seemed to suppress the growth of aggregates until 4 days of incubation, although larger amorphous aggregates with an average diameter of more than 100 nm were observed in the absence after 24 h incubation. Compared to ATP, ADP exhibited a smaller effect on the suppression of fibril formation based on the ThT assay (Fig. 2). AFM images showed fibril formation in the presence of ADP after 24 h (Fig. 3). The AFM images at higher magnification (Fig. S7 and S8) and those in the absence of Aβ (Fig. S8) suggest that these aggregates mainly consist of Aβ.

## Discussion

ATP can alter the aggregation process of Aβ in the brain since Aβ can accumulate intraneuronally and perform some functions in AD^20^, although senile plaques mainly consist of extracellular Aβ deposits. The effect of ATP at the early aggregation stage of Aβ_42_ was demonstrated by the ThT assay, which showed a gradient increase in fluorescence (Fig. 2). This suggests the aggregates formed in the presence of ATP include β-sheet structure which is distinct from those in the other conditions. However, evaluating the aggregation process at the early stage is challenging due to the instability of the aggregates of Aβ_42,_ which has a high tendency to aggregate. DXB monitors the dynamics of Aβ, and the decay constant values decrease as aggregation proceeds based on a previous report^14^. ATP increased the decay constant values immediately after the addition, suggesting that ATP alters the aggregation process from the early stage. ThT analysis showed that ATP induces aggregates to increase the fluorescence at the early stage. It is plausible that the higher dynamics prevent the formation of aggregates with lower stabilities and, as a result, induce solely stable aggregates. Since various reports have predicted hydrophobic interactions between the adenine moiety of ATP and Aβ^21,22^, ADP with an adenine moiety should also interact with Aβ to affect the aggregation process. ADP significantly increased Aβ dynamics after 1 day of incubation (Fig. 1 and Fig. S4), and ADP may partially inhibit the on-pathway.

The dynamics increased after 1 day of incubation (Fig. 1 and S4) which is probably due to changes in their formation during the fibrosis progress. CD spectrum suggests that the content of β-sheet structures continues to increase after 1 day of incubation (Fig. S6). FT-IR spectrum showed a distinct peak of spectrum in the presence of ADP after 1 day of incubation from those in the absence (Fig. S9). These results are consistent with the results of the DXB measurements, showing the significantly higher dynamics with ADP as compared to those in the absence (Fig. 1). DXB can detect the rotational fluctuation of Aβ molecules on the sub-nanometer scale. DXB analysis could be a useful method for the evaluation of the inhibitory effect of fibrillar formation by various molecules.

## Methods

### Sample preparation for DXB

Aβ was dissolved in 0.1% NH_4_OH/1 mM DMSO to a concentration of 0.1 mg/mL. ATP, ADP, and AMP (10 mM) were added to the Aβ solution and reacted at 37 °C for 0 min, 30 min, 1 day, and 4 days. Aβ solution without reagent as a control was also measured after the same reaction time at 37 °C as when reagent was added.

The immobilization of Aβ molecules onto the substrate film and the labeling of Aβ molecules with gold nanoparticles were performed based on previous studies^14^. To immobilize Aβ to the polyimide film by amine coupling, 100 μL of NHS/WCS (Dojindo) with minimum essential medium (MEM) buffer was added to the film after KOH exposure. The films were incubated at room temperature (RT) for 10 min. The Aβ solution was dissolved in activation buffer to a concentration of 70 μg/mL. Then, 100 μL of Aβ solution was added to the film and incubated at RT for 30 min. Blocking solution was then added to the film, and the film was washed with phosphate-buffered saline (PBS) buffer after 30 min of RT reaction.

In the 0 min condition, Aβ molecules were immobilized on the substrate film, and each reagent was added and measured immediately. In the 30 min condition, Aβ was immobilized on the substrate film, left to react at 37 °C for 30 min, and then measured. In the 1 day and 4 day conditions, each reagent was added to the Aβ solution, and Aβ was immobilized on the substrate film after reacting at 37 °C for the specified duration.

Gold nanoparticles for labeling Aβ molecules were prepared according to the following procedure. A 60 nm gold nanoparticle solution (Cytodiagnostics Inc.) was centrifuged at 10,000 *g* for 5 min at 4 ℃. The supernatant solution was then discarded and 50 μL of PBS was added to it. After dissolving well by pipetting, the solution was added to the film on which the Aβ molecules were immobilized.

To label Aβ with gold particles, 100 µL of *N*-succinimidyl 3-(2-pyridyldithio)propionate (SPDP) crosslinker dissolved in DMSO was added to the Aβ substrate film and reacted at RT for 60 min. Next, 10 mM EDTA with PBS and 0.3 mM dithiothreitol were added to the film and reacted at RT for 30 min to introduce thiol groups to SPDP. Gold nanoparticles (Cytodiagnostics) with a size of 40 nm were centrifuged at 3000×*g* for 10 min at 4 °C, and the supernatant solution was discarded and concentrated. To bind the gold nanoparticles and SPDP via disulfide bonds, concentrated gold nanosolution was added to the film and reacted for 120 min at RT. The film was then washed with PBS.

### Diffracted x-ray blinking

DXB measurements were conducted using the Photon Factory Advanced Ring AR-NW12 beamline. Time-resolved diffraction images were recorded using a 2D photon-counting detector (PILATUS 2 M, Dectris, Switzerland). Measurements were performed at room temperature. X-ray diffraction images were taken in a series of 2010 images with an exposure time of 50 ms. Time profiles of Au(111) intensity changes were extracted from these images using ImageJ. The time-resolved diffraction intensity in Au(111) was analyzed at each pixel by the autocorrelation function as follows:

$$I\left(\tau \right)=\frac{\langle I\left(t\right)I\left(t+\tau \right)\rangle }{\langle { I\left(t\right)}^{2}\rangle },$$

where I(t) is the diffraction intensity, the brackets <  > indicate the time-averaged value, and τ is the lag time. The ACF curves were fitted to an exponential curve by ACF(t) = Aexp(−Гt) + y, where A is the amplitude, y is the conversion factor, and Г is the decay constant. The decay constants were chosen to satisfy the following conditions: (i) 0 < y, 0 < A and 0 < Г, and (ii) residual values between the fitted and actual ACF curves of less than 1.0^23,24^. These calculations were performed for all pixels. The distribution of the decay constants of Au (111) diffraction was visualized using histograms and box plots to estimate the dynamic behavior of the protein molecules (Fig. S1C).

Because the distributions of the decay constant did not show a Gaussian distribution, the distributions were statistically analyzed with the nonparametric Wilcoxon rank sum test.

To evaluate whether the distribution of decay constants contains multiple distribution components, we clustered the distribution components by a Gaussian mixture model with the EM algorithm. The analysis was performed using R scripts. Based on the likelihood values, the distributions of decay constants were classified into three Gaussian components (Fig. S5).

### Evaluation of fibril formation

The Aβ monomer was prepared by converting iso-Aβ (Peptide Institute, Inc., Osaka, Japan) in phosphate-buffered saline (PBS) consisting of 137 mM NaCl, 2.7 mM KCl, 10 mM Na_2_HPO_4_, and 2 mM KH_2_PO_4_^17^. The Aβ monomer was preserved in dimethyl sulfoxide (DMSO) prior to use since the conversion proceeded in a few minutes. The aggregation proceeded in a final concentration of 10% v/v DMSO where the structure of Aβ_1–40_ is little affected^25^. The β-sheet structures on the surface of the assemblies of Aβ_42_ were analyzed by the ThT assay^26^. The fluorescence of 10 μM ThT in the presence or absence of 2.2 μM Aβ_42_ was monitored at an excitation wavelength of 444 nm and emission wavelength of 485 nm. The solutions were monitored every 30 min and shaken for 5 s prior to each measurement.

### Evaluation of aggregate shape

The shapes and sizes of the assemblies were observed by AFM. The solution of the assemblies (10 μL) of 0.1 mg/mL Aβ_42_ was dripped and spread onto fresh mica and dried by vacuum drying for a few minutes. Measurements were performed in alternating current (AC) mode at room temperature. The sample was measured by a typical resonance frequency of approximately 190 kHz and a spring constant of 4.5 N/m. The sizes of more than 6 assemblies were measured, and the mean and standard deviation were determined.

### Supplementary Information

Supplementary Figures.

Supplementary Table S1.

## Data Availability

The raw data for Figs. 1, S4, and S5 has been deposited in Figshare repository, 10.6084/m9.figshare.25230083. The data of Figs. 2, S6 and S9, are publicly available in Figshare repository, 10.6084/m9.figshare.25218485. The data that support the findings of this study are available from the corresponding author on reasonable request.
