# Supplementary material for: Adenosine triphosphate induces amorphous aggregation of amyloid β by increasing Aβ dynamics
Source: Sci Rep. 2024 Apr 7;14:8134. doi: 10.1038/s41598-024-58773-6 (PMC10999452; doi:10.1038/s41598-024-58773-6)
Supplement: Supplementary file 1 — Supplementary Figures. [file 41598_2024_58773_MOESM1_ESM.pptx]

## Slide 1
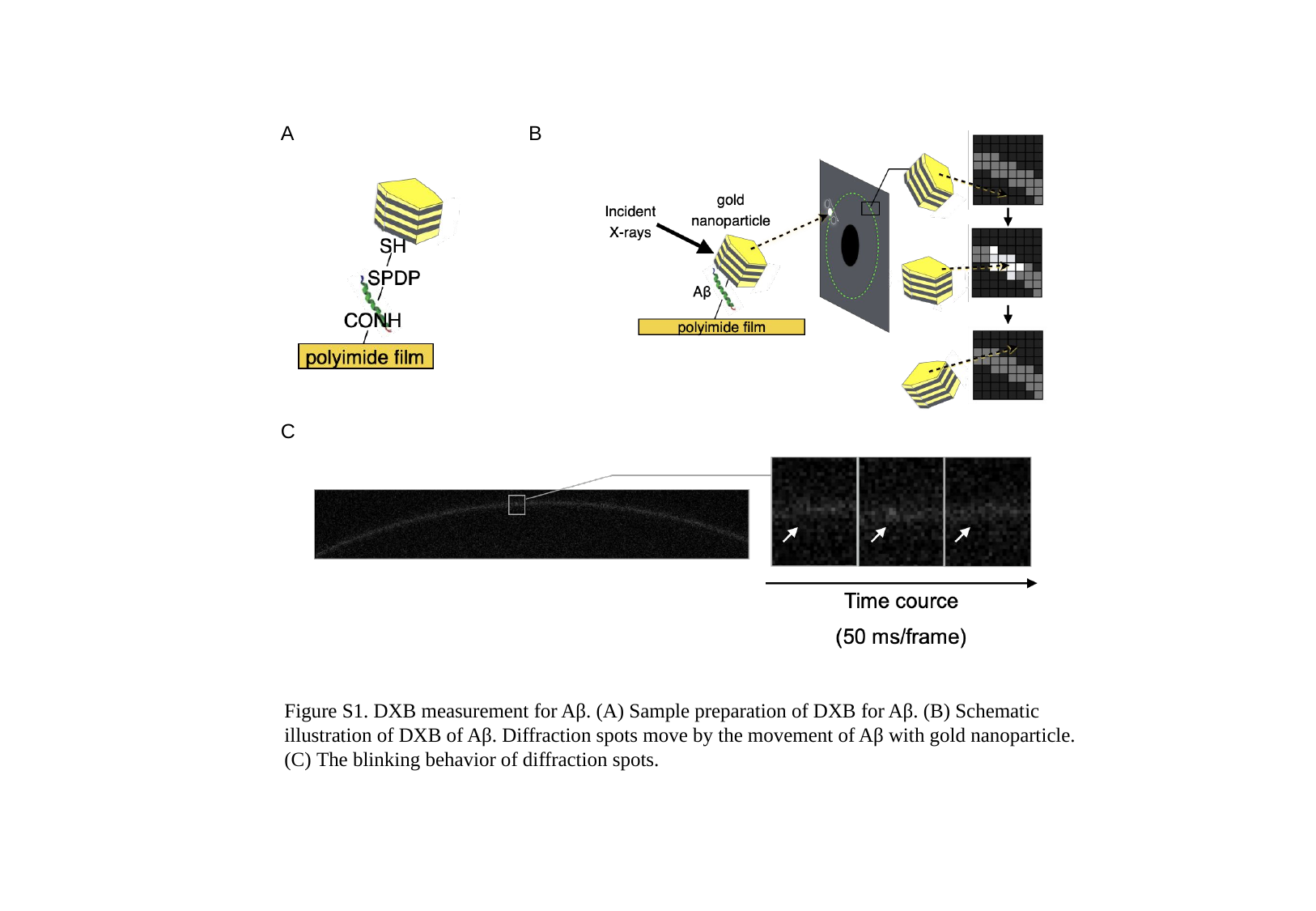

A
B
C
Figure S1. DXB measurement for Aβ. (A) Sample preparation of DXB for Aβ. (B) Schematic illustration of DXB of Aβ. Diffraction spots move by the movement of Aβ with gold nanoparticle. (C) The blinking behavior of diffraction spots.

## Slide 2
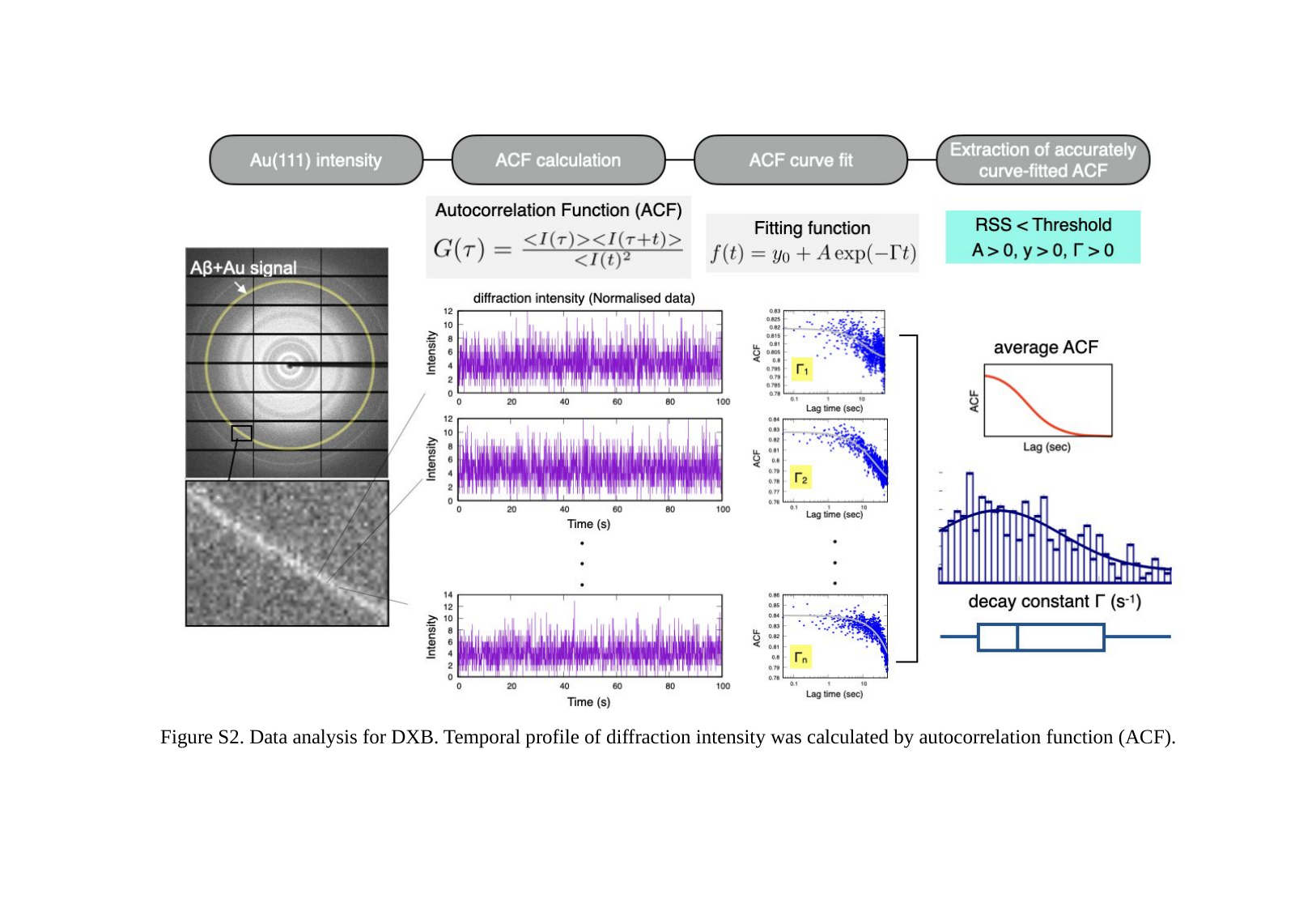

Figure S2. Data analysis for DXB. Temporal profile of diffraction intensity was calculated by autocorrelation function (ACF).

## Slide 3
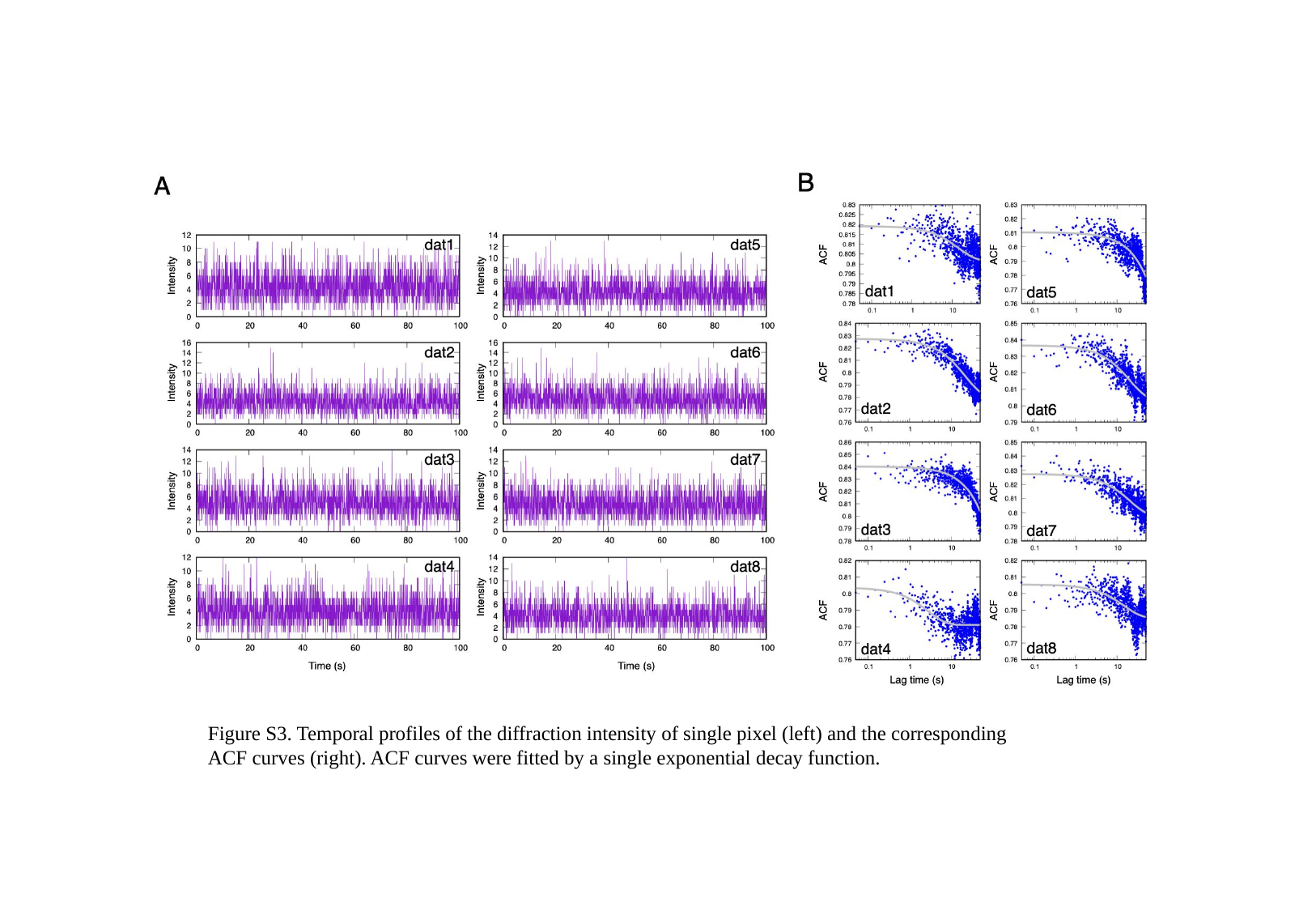

Figure S3. Temporal profiles of the diffraction intensity of single pixel (left) and the corresponding ACF curves (right). ACF curves were fitted by a single exponential decay function.

## Slide 4
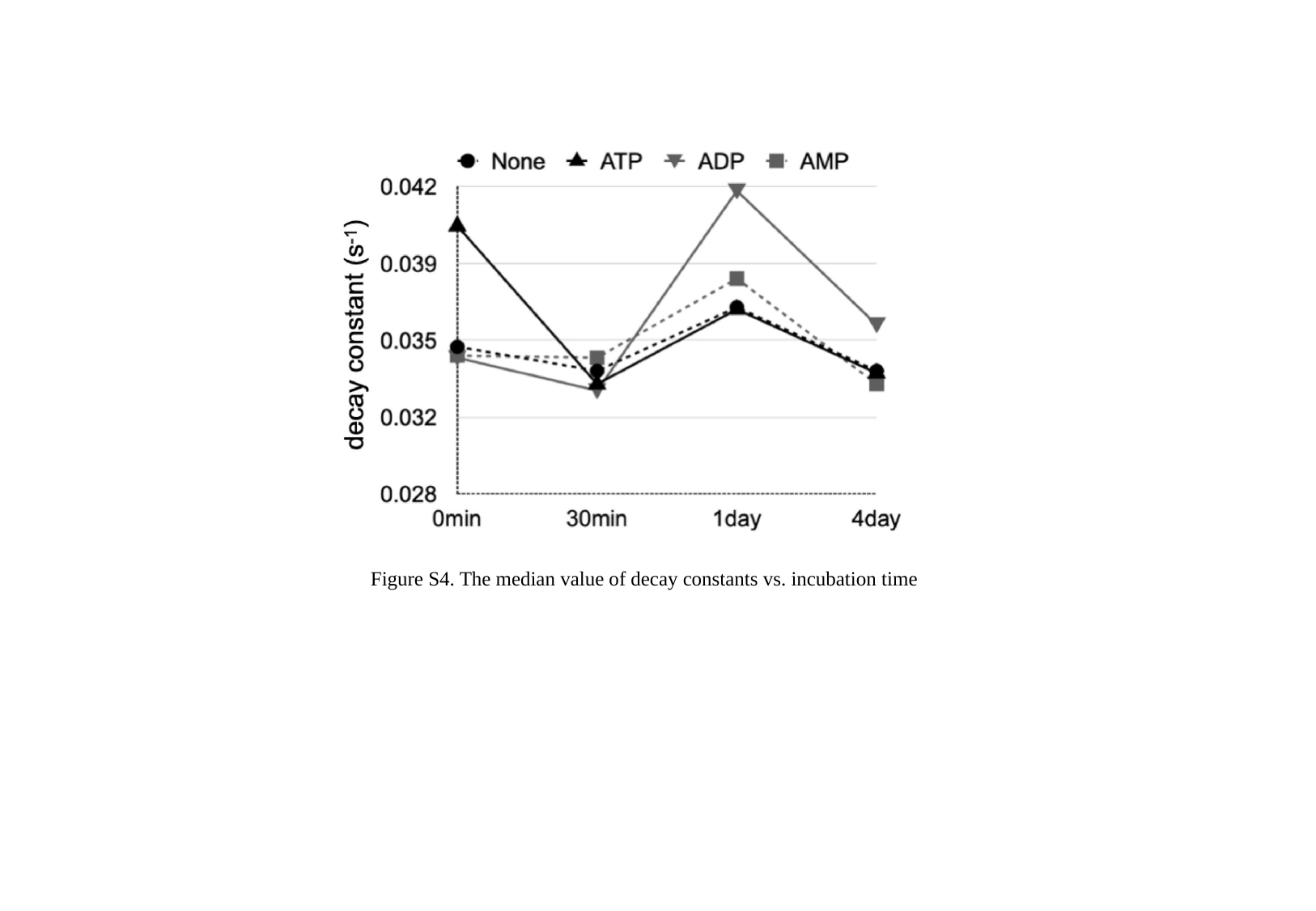

Figure S4. The median value of decay constants vs. incubation time

## Slide 5
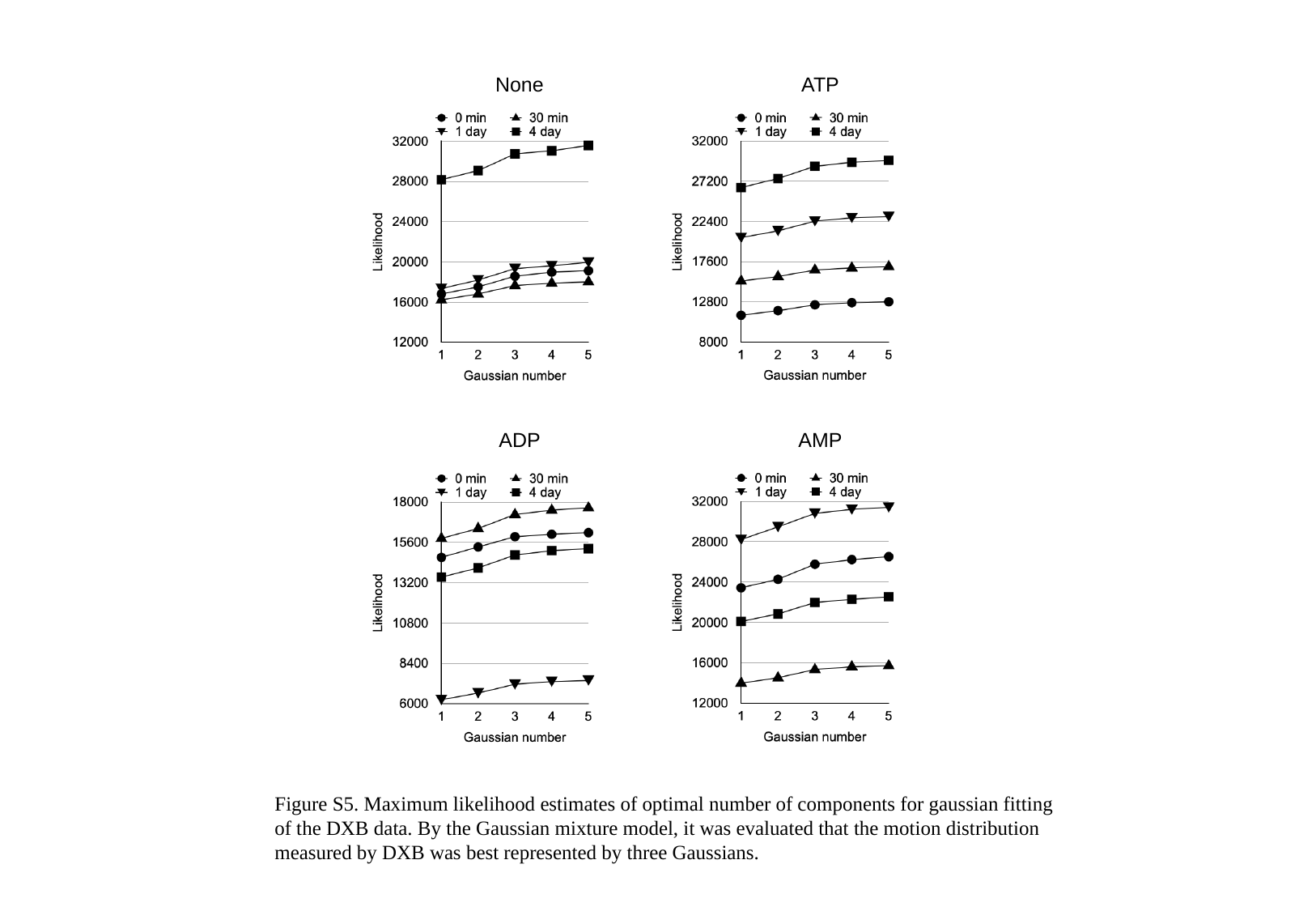

None
ATP
ADP
AMP
Figure S5. Maximum likelihood estimates of optimal number of components for gaussian fitting of the DXB data. By the Gaussian mixture model, it was evaluated that the motion distribution measured by DXB was best represented by three Gaussians.

## Slide 6
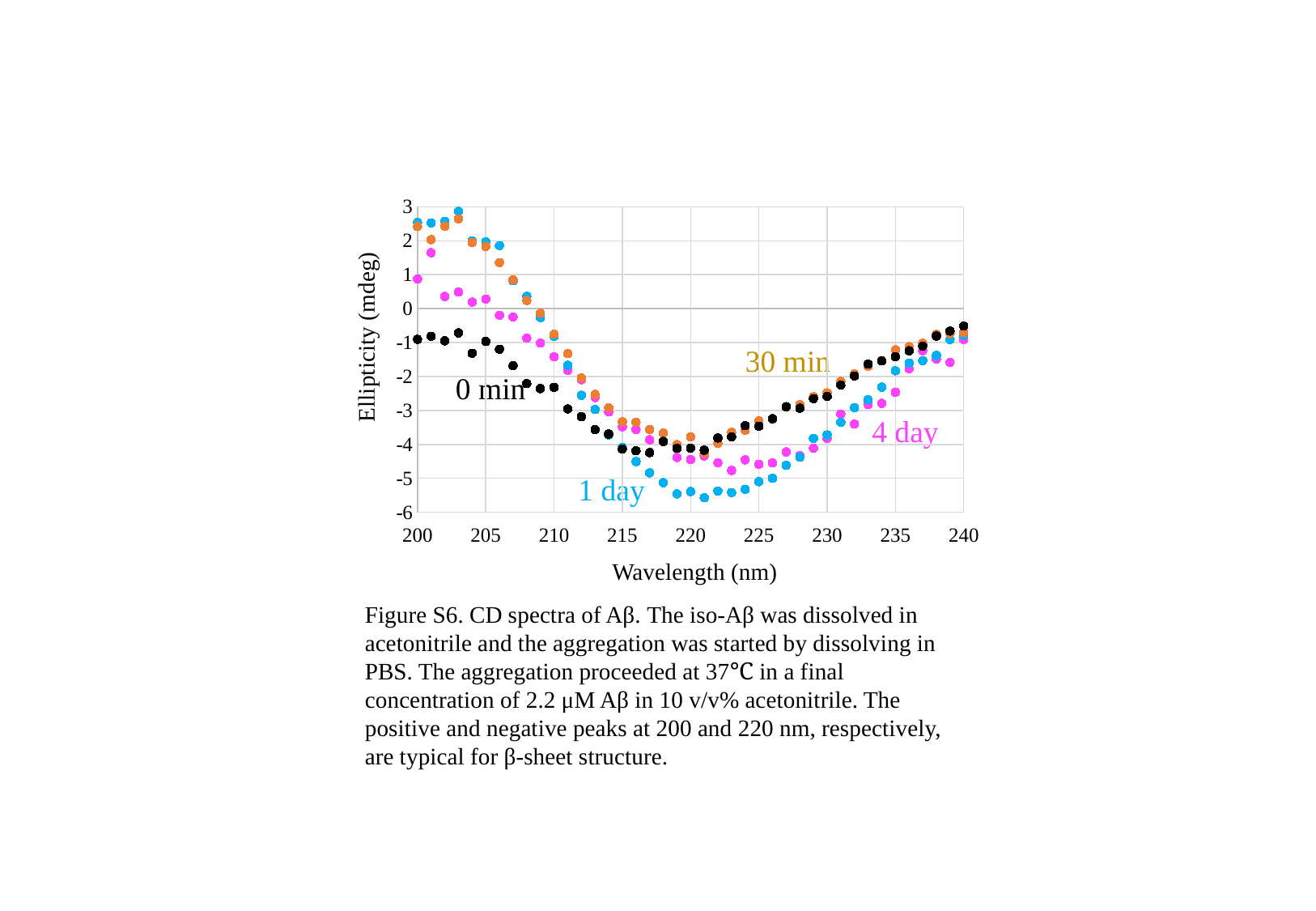

### Chart
| Category | | | | |
|---|---|---|---|---|Ellipticity (mdeg)
30 min
0 min
4 day
1 day
Wavelength (nm)
Figure S6. CD spectra of Aβ. The iso-Aβ was dissolved in acetonitrile and the aggregation was started by dissolving in PBS. The aggregation proceeded at 37℃ in a final concentration of 2.2 μM Aβ in 10 v/v% acetonitrile. The positive and negative peaks at 200 and 220 nm, respectively, are typical for β-sheet structure.

## Slide 7
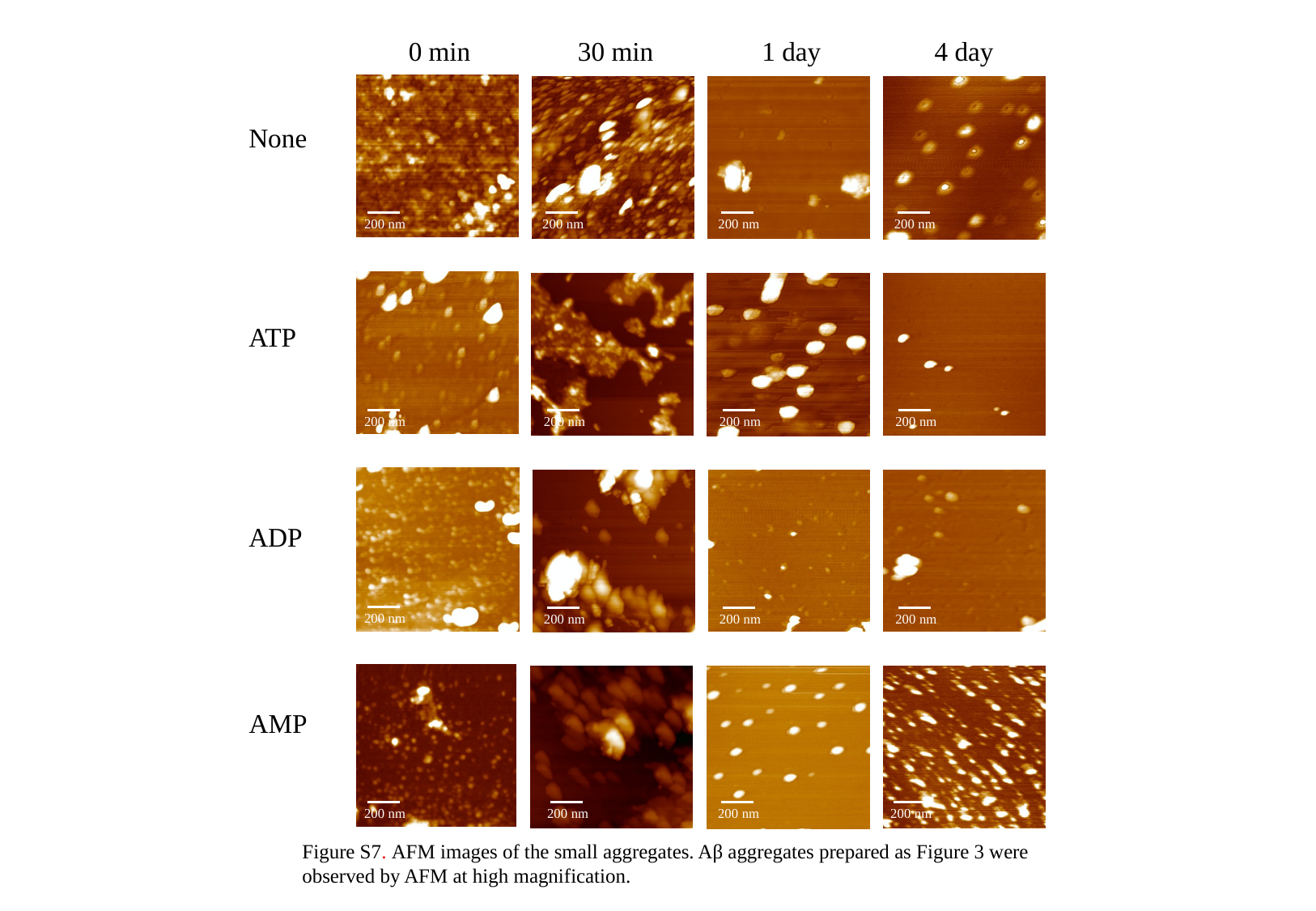

0 min
30 min
1 day
4 day
None
5 μm
200 nm
200 nm
200 nm
200 nm
ATP
200 nm
200 nm
200 nm
200 nm
ADP
200 nm
200 nm
200 nm
200 nm
1 μm
AMP
200 nm
200 nm
200 nm
200 nm
Figure S7. AFM images of the small aggregates. Aβ aggregates prepared as Figure 3 were observed by AFM at high magnification.

## Slide 8
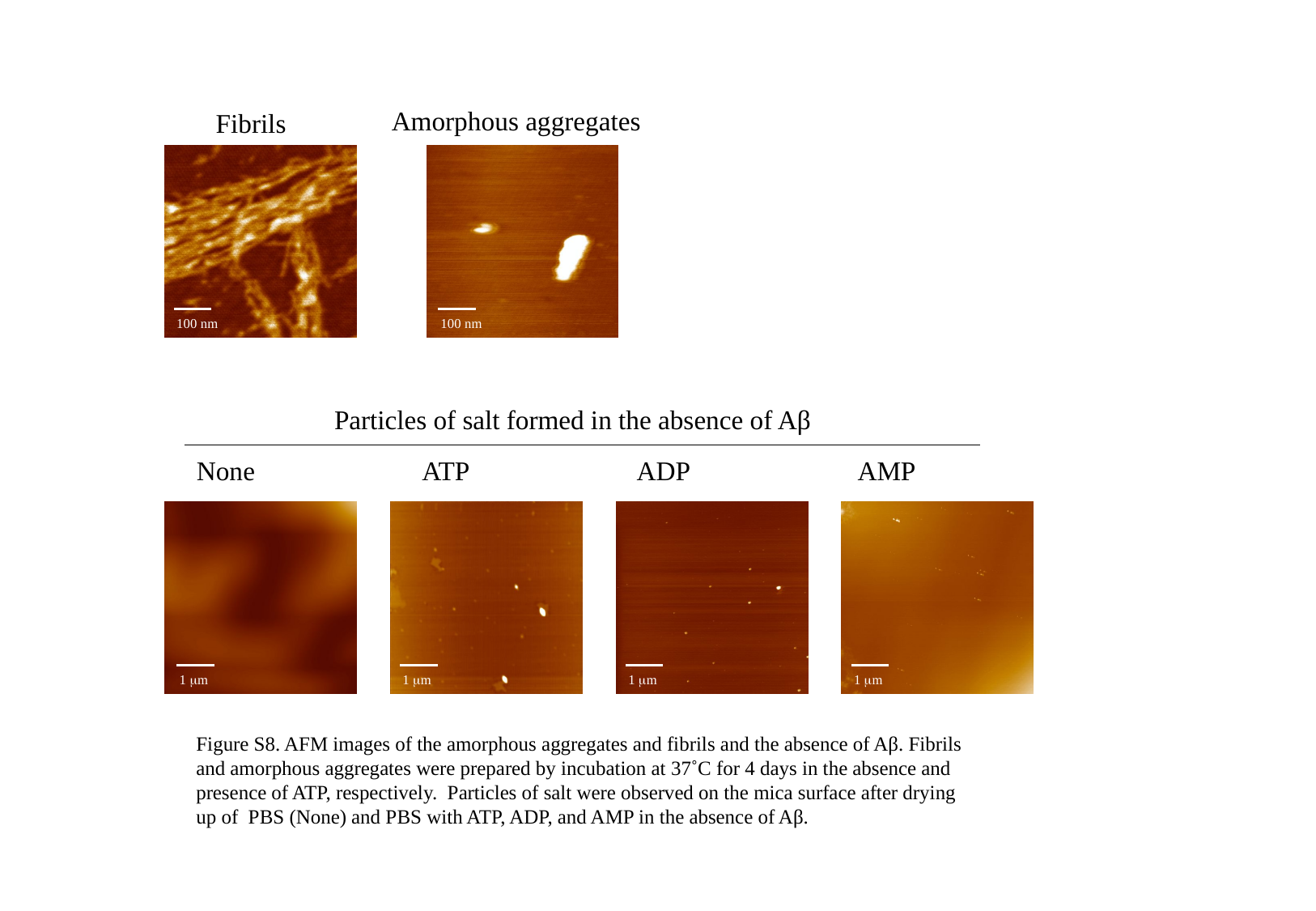

Amorphous aggregates
Fibrils
100 nm
100 nm
Particles of salt formed in the absence of Aβ
None
ATP
ADP
AMP
1 mm
1 mm
1 mm
1 mm
Figure S8. AFM images of the amorphous aggregates and fibrils and the absence of Aβ. Fibrils and amorphous aggregates were prepared by incubation at 37˚C for 4 days in the absence and presence of ATP, respectively. Particles of salt were observed on the mica surface after drying up of PBS (None) and PBS with ATP, ADP, and AMP in the absence of Aβ.

## Slide 9
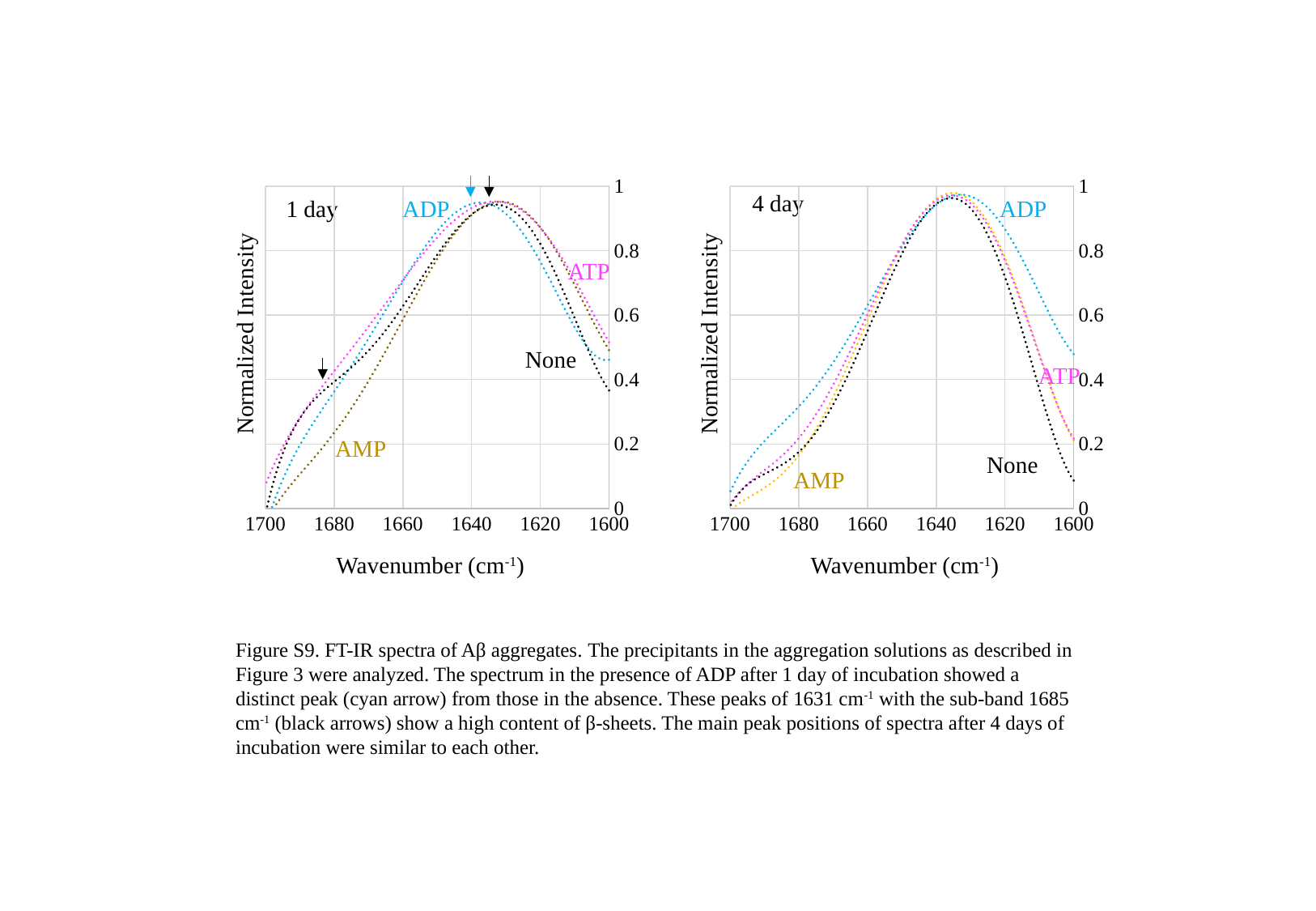

### Chart
| Category | モノマー＋PBS(1日) | モノマー＋ATP(1日) | モノマー＋ADP(1日) | モノマー＋AMP(1日) |
|---|---|---|---|---|
### Chart
| Category | モノマー＋PBS(4日) | モノマー＋ATP(4日) | モノマー＋ADP(4日)-1 | モノマー＋AMP(4日) |
|---|---|---|---|---|4 day
1 day
ADP
ADP
ATP
Normalized Intensity
Normalized Intensity
None
ATP
AMP
None
AMP
Wavenumber (cm-1)
Wavenumber (cm-1)
Figure S9. FT-IR spectra of Aβ aggregates. The precipitants in the aggregation solutions as described in Figure 3 were analyzed. The spectrum in the presence of ADP after 1 day of incubation showed a distinct peak (cyan arrow) from those in the absence. These peaks of 1631 cm-1 with the sub-band 1685 cm-1 (black arrows) show a high content of β-sheets. The main peak positions of spectra after 4 days of incubation were similar to each other.
