# Supplementary material for: Adenosine triphosphate induces amorphous aggregation of amyloid β by increasing Aβ dynamics
Source: Sci Rep. 2024 Apr 7;14:8134. doi: 10.1038/s41598-024-58773-6 (PMC10999452; doi:10.1038/s41598-024-58773-6)
Supplement: Supplementary file 2 — Supplementary Table S1. [file 41598_2024_58773_MOESM2_ESM.docx]

Table S1. Statistical analysis of the effect of ATP concentrations.

Condition Median main comp. 2nd comp. 3rd comp. q-value* *n***

0 min None 0.0338 0.0257 (63%) 0.0729 (28%) 0.2385 (8%) - 17,343

1 mM 0.0338 0.0255 (63%) 0.0773 (29%) 0.2561 (7%) 0.7827 16,003

3 mM 0.0340 0.0259 (64%) 0.0743 (28%) 0.2472 (8%) 0.3571 17,399

10 mM 0.0343 0.0246 (59%) 0.0694 (30%) 0.2274 (11%) 0.02895 15,061

Parentheses represent existence ratios in the conditions. *The q-values were calculated between the value in the absence (None) and the presence of ATP. **The number of obtained data. This analysis was calculated based on the preliminary XRD with a one-time experiment and thus showed lower intensities (approximately 4.8) than those (approximately 6.8) in Table 1 where the background intensity is approximately 4.1.
